# Spontaneous Intramuscular Abscesses Involving the Rotator Cuff Muscles in Two Cases Presenting During the COVID-19 Pandemic

**DOI:** 10.7759/cureus.11833

**Published:** 2020-12-01

**Authors:** Jamie East, Danielle Piper, Sam Chan

**Affiliations:** 1 Trauma and Orthopaedics, Queen Elizabeth Hospital, Birmingham, GBR

**Keywords:** abscess, subscapularis, supraspinatus, rotator cuff, sepsis

## Abstract

Spontaneous abscesses involving the rotator cuff muscles are a rare surgical occurrence. Patients with such abscesses are often initially misdiagnosed or there is a significant diagnostic delay. Herein, we report one case of a spontaneous intramuscular abscess involving the subscapularis muscle and a second case of an abscess involving the supraspinatus muscle. There is a multitude of predisposing risk factors to developing an intramuscular abscess formation, which includes immunodeficiency, trauma, injection drug use, concurrent infection, and malnutrition. The most significant risk factor in our cases was poorly controlled type 2 diabetes mellitus. Poorly controlled diabetes is known to cause impaired clearance of pathogens, predisposing patients to abscess formation. Both patients also delayed presenting to the hospital due to concerns surrounding the coronavirus disease of 2019 (COVID-19) pandemic.

We describe the use of a deltoid-pectoral approach to access the subscapular abscess allowing surgical drainage. The supraspinatus abscess was drained by direct incision. We advocate utilising common and familiar approaches with or without arthroscopy where possible. These cases highlight the importance of early imaging in patients presenting with the physiological signs of infection and idiopathic shoulder pain.

## Introduction

Intramuscular abscesses involving the rotator cuff complex are a rare surgical occurrence. A PubMed literature search with the search terms abscess, pyomyositis, subscapular, subscapularis, supraspinatus, revealed 14 relevant articles. There is also limited evidence available regarding the appropriate management of such patients and the optimum operative technique. Due to the paucity of cases, patients are often misdiagnosed or there is a diagnostic delay [1-3]. Herein, we describe two such cases presenting with spontaneous intramuscular abscesses involving the subscapularis and supraspinatus muscles, respectively. In both cases, the patients’ most significant risk factor for abscess formation was poorly controlled type 2 diabetes mellitus. Poorly controlled diabetes is known to cause impaired leucocyte function, predisposing patients to developing infections and subsequent abscess formation [4-5]. We describe the case of an intramuscular subscapular abscess surgically drained by a deltoid-pectoral approach and an intramuscular supraspinatus abscess drained by direct incision.

## Case presentation

Case 1: surgical drainage of a subscapularis intramuscular abscess using a deltoid-pectoral approach

Patient X, a 47-year-old left-handed female, who works in a supermarket, presented with a six-day history of acute onset of left shoulder pain. The pain was reported to be sharp in nature, 10/10 severity (on the patient-reported pain scale), and was exacerbated by multiplanar shoulder movements. She was unable to carry out her activities of daily living (ADLs) pain-free and suffered from persistent nocturnal pain. She also reported a three-day history of intermittent fevers. She had a past medical history that included obstructive sleep apnoea, polycystic ovarian syndrome, Asperger's syndrome, depression, fibromyalgia, type 2 diabetes mellitus (insulin-dependent), and hypertension. A review of her medical notes from previous admissions revealed an admission 12 months previous with a superficial abscess of the anterior abdominal wall, which was managed with surgical drainage. No complications or recurrence were noted. On further questioning, she reported poor compliance with her regular medications, including her diabetes medication. 

Blood tests on admission showed a C-reactive protein (CRP) level of 462 mg/L, white cell count (WCC) of 21.9 x 10^9^/L, neutrophils 18.4 x 10^9^/L, haemoglobin (Hb) of 133 g/L, and glycosylated hemoglobin (HbA1c) of 113 mmol/mol. Observations revealed she was pyrexial and tachycardic, but recorded blood pressure and respiratory rate were within the normal ranges. On examination, she displayed a globally reduced active and passive range of movement of the left shoulder (limited by pain) and tenderness over the posterior aspect of the shoulder joint. Attempted intra-articular aspiration of the left shoulder joint from an anterior and posterior approach was unsuccessful in yielding any fluid aspirate. 

**Figure 1:**
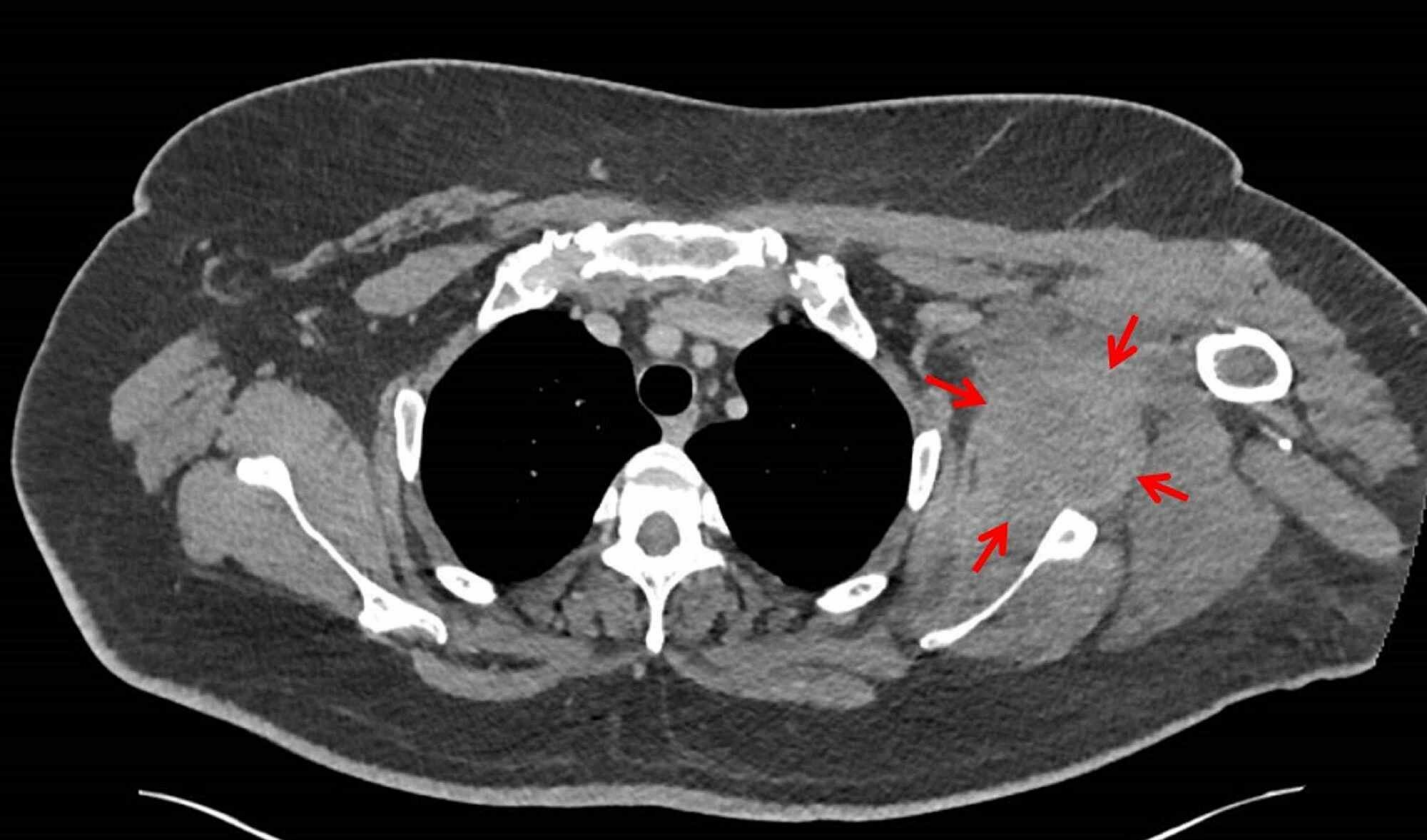
Computerised tomography scan of the thorax, axial image The red arrows illustrate the position of the subscapular abscess.

She was admitted under the care of the physicians and managed as ‘sepsis of unknown origin’ due to her displaying physiological signs of systemic inflammatory response syndrome (SIRS) but in the absence of obvious foci of infection. She was prescribed antibiotics on advice from the Microbiology team (intravenous gentamicin and co-amoxiclav). Over the following 48 hours, the patient continued to have a ‘swinging pyrexia’ and displayed signs of an ongoing SIRS response. Subsequently, contrast-enhanced computerised tomography (CT) of the thorax, abdomen, and pelvis revealed a 3.6 x 6.5 x 6.0 cm (TR x AP x CC) lobulated, low attenuation area within the left subscapularis muscle with extensive stranding in the surrounding fat, extending into the axilla with associated reactive-appearing axillary nodes (Figure 1). No bony lesion or features of osteomyelitis were identified within the scapula or humerus. There was no evidence of glenohumeral joint abnormality or joint effusion. The remaining left shoulder girdle muscles were within normal limits. The overall appearance, according to the reporting musculoskeletal radiology consultant, was suggestive of an acute intramuscular process, such as an abscess formation or haematoma. Following a multidisciplinary discussion and patient consent, it was decided to proceed with open surgical drainage of the left subscapularis intramuscular collection.

Surgical Approach

Following a general anaesthetic, the patient was set up in a supine position on a shoulder table inclined at 45°. The surgical approach was via a deltoid-pectoral approach. In this case, the cephalic vein was mobilised medially. The deltoid was dissected from the proximal humerus using blunt dissection and the conjoint tendon was retracted medially. The clavipectoral fascia appeared friable and oedematous. As the arm was brought into external rotation, the subscapularis muscle was brought into view and identified. The muscle fibres were opened by blunt dissection using MacIndoe scissors. The abscess was breached, frank pus was located, and abscess loculations were broken down. As the muscle belly of the subscapularis extends medially and inferiorly and is not easily visible, any pus located was drained using a Yankauer suction catheter. Multiple samples were obtained and sent for microbiology. The abscess was then curetted and washed with 3 litres of 0.9% saline. A 14-gauge Redivac drain was inserted into the wound and the wound was subsequently closed in layers.

Postoperatively, the patient’s arm was immobilised in a polysling for two weeks for comfort, and she was commenced on finger, hand, wrist, and elbow exercises. The patient commenced pendulum exercises as tolerated and was restricted to an active-assisted range of movement up to shoulder height and 0° external rotation for four weeks. The surgical drain was removed 48 hours postoperatively. The intraoperative samples grew *Staphylococcus aureus*, which was sensitive to both flucloxacillin and clindamycin. Patient X was prescribed a further seven days of intravenous flucloxacillin and her biochemical markers improved significantly (CRP 96, WCC 11.5) seven days postoperatively. The patient made a good recovery, experiencing no postoperative re-accumulation of the abscess or symptoms of infection. She was discharged with a further four weeks course of clindamycin on the advice of the microbiology team. 

**Figure 2:**
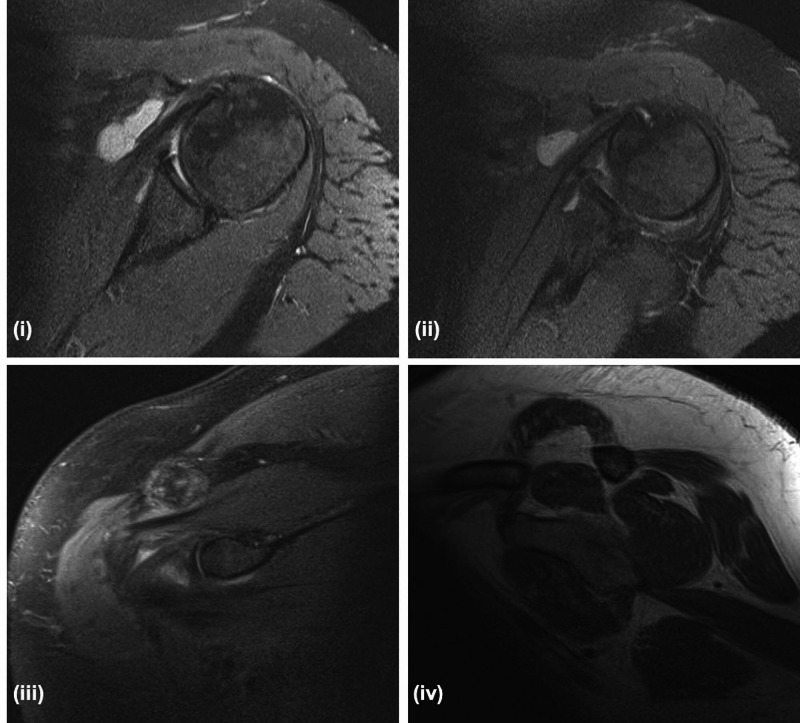
Magnetic resonance imaging (MRI) scan of the left shoulder (fat suppression-weighted) (i) axial image shows a residual subcoracoid effusion, but intact subscapularis tendon; (ii) axial image shows a residual subcoracoid effusion, and an intact subscapularis tendon and muscle belly; (iii) coronal image shows an intact subscapularis tendon and muscle belly; (iv) T1-weighted sagittal image shows a residual subcoracoid effusion and an intact subscapularis tendon and muscle belly

The patient underwent an MRI four months post-surgery as she was complaining of some residual discomfort and stiffness. Interestingly, the images showed complete resolution of the abscess, reconstitution of the subscapularis muscle fibres, and an intact tendon. The only sign of the previous infection was a subcoracoid effusion that was not clinically significant (Figure 2). 

The patient was last reviewed in the clinic six months after surgery, and there were no clinical indications of recurrence of infection. The patient has functionally improved to the point where she has gone back to work. The range of movement in her left shoulder had continued to improve; the patient was able to forward elevate to 150° and regained external rotation to 45° (comparable to contralateral side). Her internal rotation was reduced - only being able to internally rotate her hand to reach the lumbosacral junction, but it was felt that there was still time for this to improve. There was good strength in all of the rotator cuff muscles clinically. The patient recorded an Oxford Shoulder Score (OSS) of 31/48 at six months.

Case 2: surgical drainage of a spontaneous supraspinatus intramuscular abscess

A 51-year-old male (Patient Y) was brought in by ambulance to the Emergency Department as a medical alert due to symptoms of persistent vomiting and increased drowsiness, of which the history and duration were unclear. His blood glucose levels were noted to be unrecordable by the ambulance crew. He had no documented past medical history in his medical records. Initial investigations revealed a profound metabolic acidosis. Venous blood gas showed: pH 7.17, sodium (Na) 170.3 mmol/L, bicarbonate (HCO_3_) 7.7 mmol/L, lactate 4.41 mmol/L, base excess (BE) -18.8, and glucose > 33.3 mmol/L. Other biochemical markers showed a CRP of 229 mg/L, WCC 19.6 x 10^9^/L, HbA1c 138 mmol/mol, and ketones 5.9 mmol/L.

Initially, he was managed by the physicians as a mixed picture of diabetic ketoacidosis (DKA) and hyperglycaemic hyperosmolar state (HHS) with input from the endocrinology team. His physiological and biochemical parameters improved with treatment and the patient became more responsive. Subsequently, he was noted to have a tender, fluctuant right thigh mass, measuring approximately 10 cm x 15 cm in size. He was reviewed and underwent an incomplete surgical incision and drainage of the thigh abscess on the ward. Intraoperative samples isolated *Staphylococcus aureus*.

**Figure 3:**
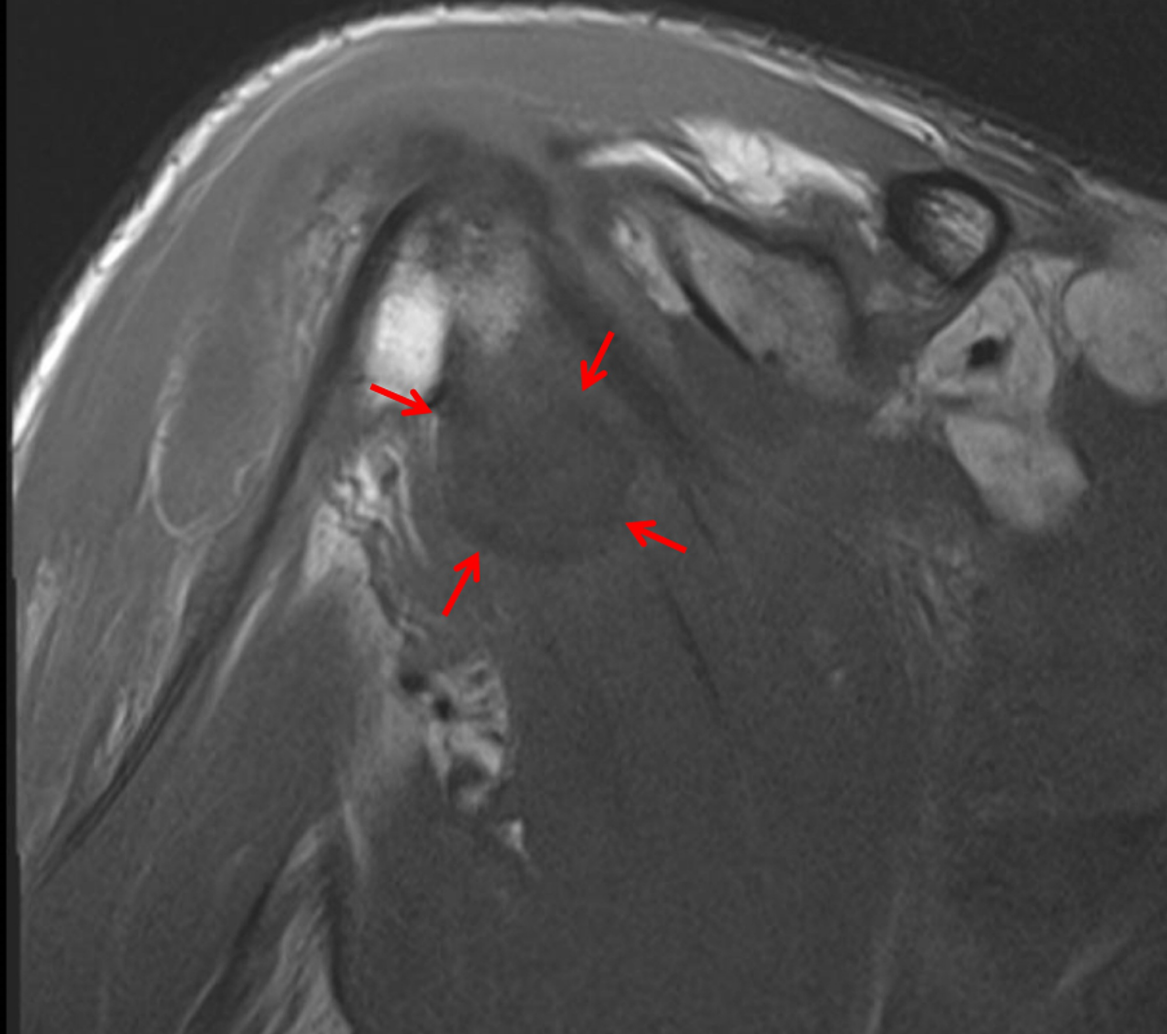
Magnetic resonance imaging (MRI) scan of the right shoulder, coronal section The red arrows indicate the position of the supraspinatus abscess.

Approximately 48 hours later, Patient Y reported the acute onset of atraumatic pain in the right shoulder. Examination revealed generalised shoulder tenderness on palpation. The range of movement of the affected shoulder was limited to 0 - 80° flexion, 0 - 80° abduction, and 0 - 10° external rotation. The joint was of a comparable temperature to the opposite side with no evidence of erythema or cellulitis. A subsequent MRI scan of the right shoulder demonstrated a 10 cm x 3.9 cm x 3.3 cm transverse x anteroposterior x craniocaudal (TR x AP x CC) fluid collection, situated within the posterior fibres of the supraspinatus muscle (Figure 3). There was also a further 1.8 cm x 0.8 cm fluid collection within the distal aspect of the subscapularis muscle, as well as extensive oedema within the anterior aspect of the deltoid muscle. Less pronounced oedema was identified within the subscapularis, supraspinatus, and infraspinatus muscles. There was extensive fluid within the subacromial/subdeltoid bursa which demonstrated extensive synovial stranding within it, in keeping with subacromial/subdeltoid bursitis.

Surgical Approach

Due to the locations of the potential collections on the imaging findings (supraspinatus +/- subscapularis muscle belly), an alternative surgical approach was proposed to address the pathology in this patient’s case. Therefore, a diagnostic shoulder arthroscopy was felt to be more appropriate to confirm the scan findings before proceeding. Following a general anaesthetic, the patient was positioned in a beach chair position on a shoulder table. Standard anterior and posterolateral portals were sited. A diagnostic arthroscopy of the subacromial space revealed no evidence of infection or pus, nor any collection in the lateral aspect of the subscapularis muscle belly. A direct incision over the supraspinatus muscle body revealed a collection of pus, which was drained. Although there was no macroscopic evidence of infection in the subacromial space, once the collection in the supraspinatus muscle was drained, it was noted to be in communication with the subacromial space. Therefore, a thorough washout of the abscess cavity and subacromial space was performed with 4 litres of 0.9% saline. The incision was closed around a surgical drain. Samples were sent for microbiology, which isolated *Staphylococcal aureus*. The Redivac drain was removed two days postoperatively. 

The patient reported some ongoing pain and stiffness of the affected shoulder over the subsequent two weeks. A repeat ultrasound scan two weeks postoperatively revealed no evidence of a recurrence of the abscess in the muscle or subcutaneous fat around the shoulder girdle. The patient was reviewed in the clinic two months post-surgery. He was noted to be pain-free in his right shoulder and had regained normal shoulder movement.

## Discussion

Abscesses are collections of pus in confined tissue spaces, usually caused by a bacterial infection. The most commonly isolated pathogen from abscesses is *Staphylococcus aureus*, as was the case in both of our patients. There are several hypothesised aetiologies that can result in abscess formation; the causative organisms may enter the tissue by direct implantation (e.g., a penetrating injury with a contaminated object) [6] or secondary to trauma [2, 7]. They may also enter tissue by direct migration from a location where there is resident flora into an adjacent, normally sterile area because natural barriers have been disrupted (e.g., an area of cellulitis). It is also known that dissemination of infection can occur via lymphatic or haematogenous routes, leading to abscess formation at a distant site.

There are a multitude of predisposing factors to developing pyomyositis and intramuscular abscess formation, which include immunodeficiency, trauma, injection drug use, concurrent infection, and malnutrition. The most significant risk factor consistent in both our cases was poorly controlled diabetes mellitus. Patients with diabetes are known to have impaired leucocyte function, and the metabolic abnormalities of diabetes lead to inadequate migration of neutrophils and macrophages to areas of infection, along with reduced chemotaxis [4-5]. This predisposes patients to develop infections as it reduces the effective clearance of pathogens. The blood test results of our cases showed serum HbA1c levels of 113 and 138, respectively, (normal: < 42 mmol/mol), indicating longstanding, very poorly controlled diabetes in both cases. Patient Y also had a significant antecedent infection with a large thigh abscess which, due to the high bacterial load, was likely to have spread to the shoulder girdle via the haematogenous route, forming an intramuscular abscess. 

Delayed presentation due to the COVID-19 pandemic is another important factor to consider. Both patients delayed presenting to the hospital due to concerns surrounding the pandemic. This could have led to them developing a higher bacterial load of infection, increasing the chance of infection seeding to other locations. Patient X’s diagnosis of autism likely contributed to a further delay in presentation.

The infrequent nature of intramuscular abscesses of the shoulder girdle invariably means that there is often a delay in diagnosis, as was the case for both our patients. This can lead to a delay in treatment and/or inappropriate treatment and can result in avoidable harm to patients [8]. In our hospital, it is common to request an ultrasound as the first line to exclude a septic shoulder joint. As demonstrated, the uncommon locations of these abscesses can be missed and give a false reassurance that there is no abnormal pathology in the shoulder. Therefore, in these cases, it is important to highlight the lower threshold to re-image early (if symptoms persist) using MRI or CT. This may be especially true in patients with underlying risk factors for developing infections who present with sepsis of unknown origin and concurrent shoulder pain. More detailed imaging also allows delineation of the site of infection and helps avoid procedures that may seed infection to other parts of the anatomy, particularly the glenohumeral joint.

It is a well-established practise that management of abscesses involves surgical drainage and administration of antibiotics. Antimicrobial drugs alone are usually ineffective without drainage. When deciding on the type of approach, it is important to identify the location of the pathology and to have a strategy for accessing these sites. We advocate using a familiar and common approach where possible. In the first case, we describe a standard deltopectoral approach for access to the subscapular region. We felt that this would allow adequate access to the abscess and accepted that it would not be possible to visualise the whole subscapular area without extensile measures, such as a coracoid osteotomy and its incumbent risks to the musculocutaneous nerve. We note that multiple approaches to access the subscapular space have been described. Patel et al. and Furuhata et al. both utilised the same approach to access the subscapular space [7, 9]. Furuhata et al. also subsequently describe accessing the dorsal side of the subscapularis muscle belly to reduce the risk of neurovascular injury and pneumothorax [9]. The benefit of the deltoid-pectoral approach is that it enables access to both the subscapular space and glenohumeral joint. Infections involving the glenohumeral joint may not always be apparent on imaging. Cristman-Skieller et al. described a posterolateral approach as the most direct for accessing the area of infection in their case, as the bulk of the abscess was at the lateral aspect of the scapula [10]. This approach also doesn’t require any muscle detachment and is attractive as any drain insertion would be aided by gravity to minimise the risk of accumulation. However, the unfamiliar approach risks iatrogenic injury to the thoracodorsal and subscapular nerves. Saxena et al. and Nowinski et al. both describe a posteromedial approach to the subscapular space with an incision over the medial border of the scapula and dissecting the rhomboid muscle or dividing the trapezius muscle to gain exposure from the medial side [11-12]. Again, the advantages of this approach largely depend upon the location of the abscess.

The second case highlights the strategies required for addressing potential infected spaces, with particular reference to the glenohumeral joint. A missed septic arthritis would be disastrous in any setting. However, arthroscopy is a useful tool in the armamentarium for confirming scan findings and accessing hard-to-access areas.

The appropriate operative procedure is dependent on a multitude of factors, including the site of infection, intra-articular involvement, patient factors, and surgeon experience. We advocate utilising common and familiar approaches with or without arthroscopy where possible. The deltopectoral approach is a shoulder surgeon’s workhorse approach and also provides access to the glenohumeral joint where indicated. 

## Conclusions

Spontaneous abscesses affecting the rotator cuff complex are rare. There are often delays in diagnosis, which increases the risk of mortality. Early MRI or CT imaging should be considered in patients presenting with physiological signs of infection and idiopathic shoulder pain. Risk factors for developing an infection, such as poorly controlled diabetes mellitus, should be considered. The deltopectoral approach is effective in accessing the subscapular region in this case. However, the surgical approach should be tailored to the patient and guided by imaging with consideration of whether there is a concurrent intra-articular infection and preventing seeding of infection locally to other parts of the anatomy.
